# An improved method for the isolation and identification of unknown proteins that bind to known DNA sequences by affinity capture and mass spectrometry

**DOI:** 10.1371/journal.pone.0202602

**Published:** 2018-08-23

**Authors:** Pooja Murarka, Preeti Srivastava

**Affiliations:** Department of Biochemical Engineering and Biotechnology, Indian Institute of Technology, New Delhi, India; Consiglio Nazionale delle Ricerche, ITALY

## Abstract

Transcription of a gene can be regulated at many different levels. One such fundamental level is interaction between protein and DNA. Protein(s) binds to distinct sites on the DNA, which activate, enhance or repress transcription. Despite being such an important process, very few tools exist to identify the proteins that interact with chromosome, most of which are *in vitro* in nature. Here, we propose an *in vivo* based method for identification of DNA binding protein(s) in bacteria where the DNA-protein complex formed *in vivo* is crosslinked by formaldehyde. This complex is further isolated and the bound proteins are identified. The method was used to isolate promoter DNA binding proteins, which bind and regulate the *dsz* operon in *Gordonia* sp. IITR 100 responsible for biodesulfurization of organosulfurs. The promoter binding proteins were identified by MALDI ToF MS/MS and the binding was confirmed by gel shift assay. Unlike other reported *in vivo* methods, this improved method does not require sequence of the whole genome or a chip and can be scaled up to improve yields.

## Introduction

DNA binding proteins play a crucial role in transcription, DNA replication, repair, recombination and various other cellular activities [1]. To completely understand these fundamental biological processes, it is important to have knowledge about the proteins whose interplay leads to the complex control [2]. In the past, methods such as electrophoretic mobility shift assay (EMSA) [3, 4], pull down assay etc [5] have been reported to identify DNA binding proteins. These methods are mainly associated with *in vitro* DNA-protein interactions wherein a purified protein or a crude extract of protein is incubated with labeled DNA. DNA-binding proteins have specific or general affinity for DNA sequences. Some proteins involved in transcriptional regulation or other varied function may bind weakly to the DNA, hence making their isolation and identification difficult. For example, in gel shift assay, the conditions need to be optimized to get an optimal binding [6]. Similarly, in an *in vitro* pull-down assay the DNA fragment is immobilized and the crude extract is allowed to pass through. Thus, the binding conditions *in vitro* may differ with the binding conditions *in vivo*. Hence, isolation of all DNA binding proteins is not possible. Another widely used method for identifying DNA binding protein is chromatin immunoprecipitation (ChIP) assay [7, 8]. ChIP assay is used for genome wide profiling of DNA-binding proteins, histone modification or nucleosomes. Besides cost and availability, ChIP has its own technical limitations. This method requires antibodies specific to the protein of interest and therefore, it cannot be used when the proteins are not known [8]. Also, both ChIP and EMSA have low throughput, which makes these two techniques not suitable for identification of unknown factors that bind to DNA [8].

A SILAC (stable isotope labeling by amino acid in cell culture) based method was described by Ong et al., 2002 [9] for proteome analysis which was further improvised by Mittler et al., 2008 [10] to study proteins that interact with DNA. It is an *in vitro* method in which cells are allowed to grow in presence of labeled amino acids. The protein extract of these cells is passed through column containing immobilized DNA fragment. The DNA fragment is designed in such a way that it consists of a restriction enzyme site which is used for elution of proteins. The peptides generated after in gel digestion of the eluted protein are identified by mass spectrometry in combination with isotope coded affinity tag (ICAT) technology. A major limitation with SILAC based method is that it requires auxotrophy of amino acids employed for labeling, therefore this method cannot be extensively used in all types of bacterial cultures [11]. The elution of proteins is based on restriction digestion, therefore designing of DNA fragment is little tricky. There is a possibility that the restriction site is not accessible to the enzyme for digestion if a large complex is bound to DNA due to steric hindrance.

Butala et al., proposed another *in vivo* method for identification of proteins [12]. The method involves cloning the DNA of interest along with lac operators flanked by ISceI endonuclease sites. The lacI fused to FLAG tag was used for isolating the DNA protein complex. The method is very useful but it requires cloning of the desired DNA in a special type of vector, which has ISceI sites and also requires expression from ISceI meganuclease.

Dejardin and Kingston [13] proposed a method called Proteomics of Isolated Chromatin segment (PICh) for identifying the proteins associated with telomeres. The major limitation of PICh is that it cannot be used for DNA sequences that are present in single copy or are low in number in the genome. Another drawback of PICh is that it requires large amount of starting material (several hundred litres of culture).

There are only limited reports of methods that can be used to isolate the proteins that bind *in vivo* with the knowledge of short sequences of DNA. One such example of a known short DNA fragment is the *dsz* promoter of desulfurizing organisms. The genes for biodesulfurization are present in the form of an operon under the control of this promoter. The sequence of this promoter has been identified but the proteins that bind to this promoter have not been reported.

In this study, we describe a method for isolation and identification of transcription factors that bind to DNA inside the cell. It involves cross-linking of the proteins to *dsz* promoter DNA with formaldehyde when the cells are present in the log phase followed by harvesting and sonication of the cells. The promoter DNA is then subjected to digestion with exonuclease to generate overhangs, which further binds to specific biotinylated primer attached to streptavidin beads. Bound proteins are eluted based on pH. The eluates from the column are run on SDS-PAGE gel, and the protein bands, which are observed, are in gel digested and analyzed by MS/MS for identification. By this method we were able to isolate and identify transcriptosome complex consisting of 7 proteins whose interaction was evident by the interactome analysis.

## Materials and methods

### Media and growth conditions

*Gordonia* sp. IITR100 (MCC2877) [14] was cultured in minimal media. The composition of Minimal salt medium per litre was: Na_2_HPO_4_ (2.0 g), KH_2_PO_4_ (1.0 g), ammonium oxalate (4.25 g), MgCl_2_ (0.4 g) and sucrose (50 mM). Trace elements composition for 1 L was: KI (0.05 g), LiCl (0.05 g), MnCl_2_.4H_2_O (0.8 g), H_3_BO_3_ (0.5 g), ZnCl_2_ (0.1 g), CoCl_2_.6H_2_O (0.1 g), NiCl_2_.6H_2_O (0.1 g), BaCl_2_ (0.05 g), (NH_4_)_6_ Mo_7_O_24_. 2H_2_O (0.05 g), SnCl_2_.2H_2_O (0.5 g), Al (OH)_3_ (0.1 g). All the chemicals were dissolved in double distilled water. The medium was supplemented with 3 mM sodium sulfate as sulfur source. Single colony is used to inoculate 50 ml medium and incubated at 30°C and 180 rpm for about four days after which the OD_600_ was 1.

For cloning and expression studies, *Escherichia coli* (*E*. *coli*) DH5α and BL21 (DE3) and BL21 (DE3) pLysS cells were used respectively. *E*. *coli* was inoculated in LB media, incubated at 37°C at 180 rpm and the antibiotic used was kanamycin in the concentration of 50 μg/ml where required.

### Cross linking of DNA-protein complexes

For crosslinking of DNA protein complex, formaldehyde was used. Three different concentrations (0.5, 0.75 and 1%) of formaldehyde were used. After incubating at 25°C for 10 min, formaldehyde was quenched by addition of Tris (final concentration 250 mM, pH 8) and incubated at RT (25°C) for 10 min. As a control experiment, in one set of culture, the cross-linking was reversed by addition of Rapigest, 0.5 M β-mercaptoethanol and Tris (250 mM, pH 8.8), incubated at 99°C for 25 min. Four different concentrations of Rapigest 0.5, 0.75, 1 and 2% were used.

Cells from both the cultures (cross-linked and reversal of cross-linked) were harvested at 3,500 rpm for 10 min at 4°C. Supernatant was discarded and the pellet was resuspended in 1 ml sonication buffer which consisted of 20 mM Tris-Cl, 150 mM NaCl, 0.1mM PMSF, 1mM DTT, 0.1mM EDTA and 10% glycerol, pH 7.5. Sonication was performed on Q125 sonicator (Qsonica sonicators, USA) by giving 20 sec bursts and 30 sec rest for 5 min at an amplitude of 30%. After sonication, the cellular debris is removed by centrifugation at 12,000 rpm for 20 min at 4°C. The supernatant was collected in a fresh eppendorf tube and used for further experiments. The remaining was stored in -20°C.

### Isolation of DNA-protein complexes

Supernatant (88 μl) prepared above was used for performing digestion with T5 exonuclease. T5 exonuclease, 2 μl (0.2unit/μl) enzyme was added to the above supernatant and incubated at 37°C for 45 min. The activity of the enzyme was stopped by EDTA (final concentration 10mM). A control experiment was set without treatment of supernatant with T5 exonuclease. This mixture of about 100 μl DNA-protein complex is mixed with streptavidin solution containing biotinylated oligonucleotide and incubated for 30 min in a rotating wheel in cold room. To attach the biotinylated oligonucleotide to the streptavidin beads, 5’ biotin labeled PS19 primer (5 μM) (5’ CAGTCATATGCGCGTATGTGTCCTCTAACCGTAAATAGCG 3’) was attached to 200 μl streptavidin solution in a microfuge tube and incubated in a rotating wheel for 10 min at room temperature. The primer used is 40 bp in length and specific to the promoter of our interest. As a control beads without bound oligonucleotides were used.

The mixture was centrifuged at 12,000 rpm for 2 min. at 4°C and the supernatant was collected. The supernatant should contain unbound proteins. The DNA protein complex bound to streptavidin beads was washed with 200 μl binding buffer three to four times by repeated incubations in a rotating wheel for 5 min. each in the cold room. The binding buffer consisted of 12% glycerol, 12 mM HEPES (pH 7.9), 4 mM Tris-Cl (pH 7.9), 60 mM potassium chloride, 1 mM EDTA, 1 mM DTT. The bound proteins were eluted by elution buffer and four fractions each of 200 μl were collected. The elution buffer consisted of 12% glycerol, 20 mM Tris-Cl (pH 5.8), 1 M potassium chloride, 5 mM magnesium chloride, 1 mM EDTA, 1 mM DTT, 20 μg/ml BSA.

### SDS-PAGE separation, in gel digestion and identification of proteins

The eluates obtained in pull down assay were analyzed on 12% SDS-PAGE gel with 5% stacking gel [15]. The gel was silver stained and the different bands obtained in the elution lane of the gel were cut and in-gel digested with trypsin following the protocol described by Bruker Daltonics, Bremen, Germany adapted from Shevchenko et al., 1996 [16]. The digested peptides were dried in speed vac and the samples were analyzed by MS/MS on ABI Sciex 5800 TOF/TOF system, USA.

The parameters set for the MS/MS analysis were as follows: Fixed modifications: Carbamidomethyl (C), Variable modifications: Deamidated (NQ), Oxidation (M), Peptide Mass Tolerance: ± 150 ppm.

To determine the specificity of the method, a non-specific DNA, biotinylated primer of kanamycin promoter was used. The remaining procedure was identical as was performed with primer of *dsz* promoter. The elution fractions were analyzed on an SDS-PAGE and identified by MALDI-ToF MS/MS analysis.

### Recombinant DNA methods

The methods followed for cloning were according to protocol mentioned in Sambrook and Russell [17]. The gene for XRE (Xenobiotic response element) family transcription regulator was cloned between the restriction sites NdeI and HindIII in the vector pET29a. This recombinant plasmid DNA was transformed to expression strain for studying overexpression of XRE. The restriction enzymes, T4 DNA ligase, Taq DNA polymerases were purchased from New England Biolabs, USA. IPTG and other chemicals used were of molecular grade.

#### Cloning and expression of XRE family transcription regulator (Xenobiotic response element) family transcription regulator

The gene encoding for XRE like protein was amplified using primer pairs 5’ CTAG*CATATG*GTGAGCGAGCAACGCCGAATCGGGTACCAC 3’ (XRE-F) and 5’ CTAG*AAGCTT*CGTGGTCGTGCCATCGTCACCATCGCTGCG 3’ (XRE-R). The amplified PCR product was digested with NdeI and HindIII and cloned in pET29a vector between NdeI and HindIII. The vector so constructed was named pPM4. The recombinant plasmid was transformed to expression strains BL21 (DE3) and BL21 (DE3) plysS for overexpression of the desired protein. Single colony of the transformed cell was inoculated in LB media containing kanamycin (50 μg/ml). The cells were induced with IPTG (final concentration 1 mM) at an OD_600_ ~0.5, and the samples were collected at different time intervals (5 hrs and overnight after induction) and analyzed on a 12% SDS PAGE gel to check the expression.

### Purification of XRE family protein

To validate the binding of XRE protein to the *dsz* promoter, the protein was purified partially and EMSA was performed. For purification, a single colony of the BL21 (DE3) plysS cells harboring the plasmid containing the gene encoding for XRE protein was inoculated in 200 ml of LB media and induced for overexpression with 1mM IPTG. After 5 hrs of induction, the cells were harvested by centrifugation at 3500 rpm for 10 min. The cells were resuspended in 4 ml denaturing buffer A (100 mM Na_2_PO_4_, 10 mM Tris, 8 M urea, pH 8) and sonicated on Q125 sonicator (Qsonica sonicators, USA) by giving 30 sec bursts and 20 sec rest for 5 min at an amplitude of 25%. The cell debris was removed by centrifugation at 10,000 rpm for 15 min and the supernatant was collected in a fresh tube. The sonicated supernatant was added to the Ni-NTA column equilibrated with the buffer A. The suspension was incubated at 4°C on a rotating wheel for 2 hrs to allow the binding of the protein to Ni-NTA agarose resins. The flow through was collected and the column was washed with buffer B (100 mM Na_2_PO_4_, 10 mM Tris, 8 M urea, pH 6.5). The proteins bound to the column was eluted by elution buffer C and D (100 mM Na_2_PO_4_, 10 mM Tris, 8 M urea, pH 5.5 and 100 mM Na_2_PO_4_, 10 mM Tris, 8 M urea, pH 4.5 respectively). All the different collected fractions were run on 12% SDS PAGE gel to check the fraction which contains the protein of our interest. The elution fraction with buffer C (contained the XRE protein) was renatured by adding the eluate (5 ml) dropwise to the 50 ml renaturation buffer (10% glycerol, 25 mM Tris-Cl and 150 mM sodium chloride, pH 8) in cold room by stirring continuously with magnetic stirrer. The renatured protein solution was further dialyzed overnight against the renaturation buffer with 2–3 changes to remove the traces of urea. The renatured sample was run on 12% SDS PAGE gel to check the purity of the protein.

### EMSA with partially purified XRE

EMSA was performed to determine the binding of XRE with the *dsz* promoter. For this purpose, *dsz* promoter was Cy5 labeled by PCR amplification using Cy5 PS19 primer (5’ CAGT*CATATG*CGCGTATGTGTCCTCTAACCGTAAATAGCG 3’). Reaction mixture consisting of Tris binding buffer (20 mM Tris Cl, 5 mM MgCl_2_, 0.1 mM EDTA, 6% sucrose, 100 mM KCl and 1 mM DTT, pH 7.5), poly d(I-C), poly L-Lysine, 12 ng of labeled promoter and increasing concentration of partially purified protein (5–20 μg) was incubated for 4 hrs at 4°C and was then run on 6% native TBE gel for 2 hrs at 100V. The gel was then scanned in a fluorimager (GE typhoon 9000) to see the shift. Two types of controls were used. First contained all the components of the reaction mixture mentioned above expect the protein and the second reaction mixture consisted of tris binding buffer, poly d(I-C), poly L-Lysine, 12 ng of Cy5 labeled *dsz* promoter and 20 μg of renatured washing eluate from purification (it contains other non-specific proteins present in the partially purified eluate but the protein of our interest in negligible amounts (beyond detection limit)). The reaction mixture was incubated for 4 hrs at 4°C and was then run on 6% native TBE gel for 2 hrs at 100V. The gel was then scanned in a fluorimager (GE typhoon 9000) to see the shift.

## Results and discussion

**Fig 1:**
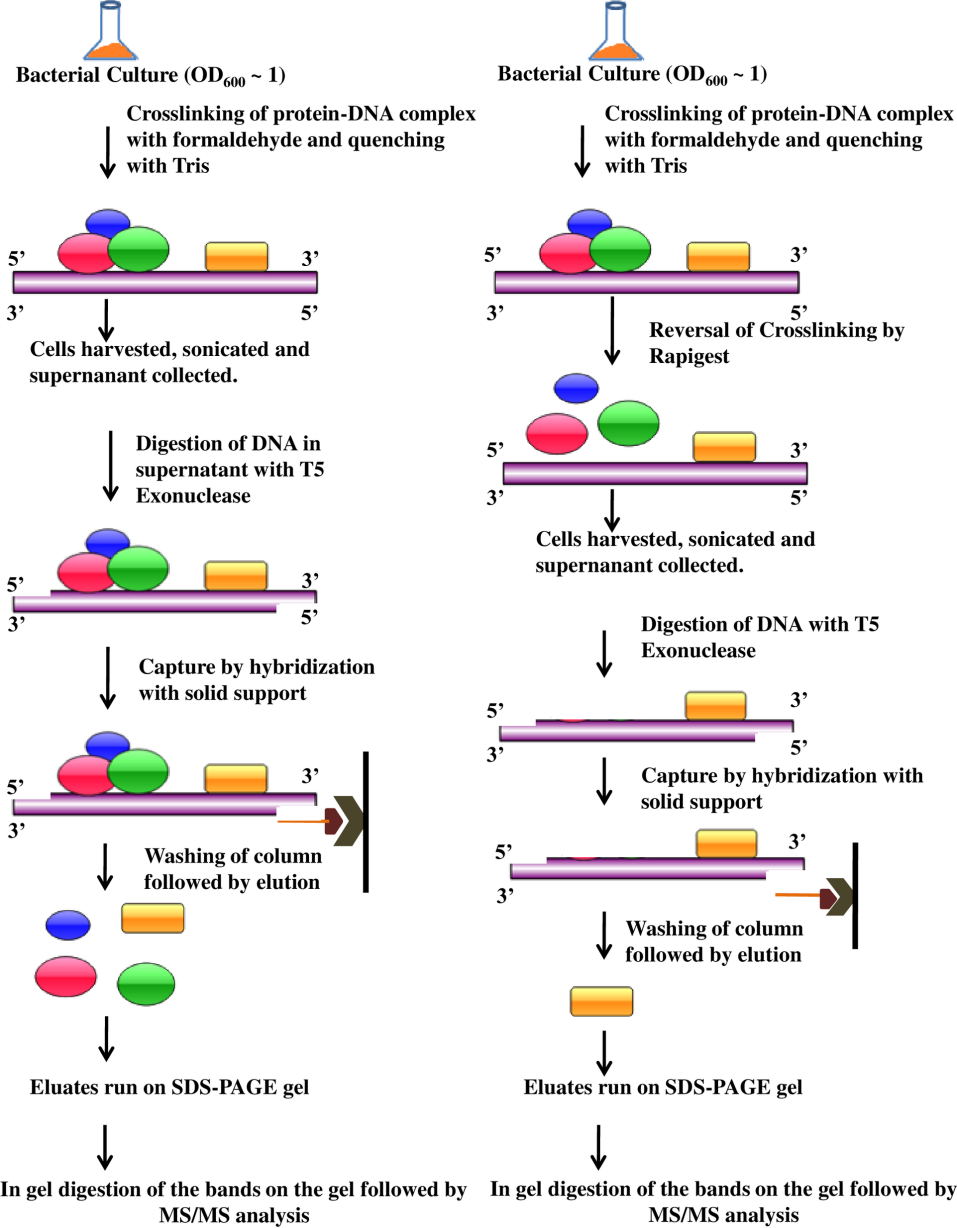
Schematic diagram showing the experimental procedure followed. The left panel shows the experiment procedure without reversing the crosslinking between DNA and the protein. In the right panel shows the method followed for control where the cross linking is reversed with Rapigest before harvesting the cells.

To mimic *in vivo* conditions outside the cellular environment is complicated thereby making it difficult for all the DNA binding proteins bind to their respective DNA. Here, we propose an *in vivo* method for the isolation of the DNA binding proteins. Our improved method for isolation of DNA binding proteins is based upon the method originally proposed by Wang, 2009 [18], modified by Wu et al. [19]. The method differs in the following aspects. 1) We propose to use T5 exonuclease instead of exonuclease III. T5 exonuclease chews the DNA from 5’-3’ direction whereas exonuclease III chews from 3’-5’ direction. In any promoter DNA, regulatory elements bind to either the 3’ end or the upstream region or both. The extra sequences at the 5’ end can be taken for the isolation of all the promoter DNA binding proteins. In this way, it is likely that the user would not miss out any protein. [20], 2) biotinylated oligo is used for pairing with the DNA bound protein complex. 3) After elution, the eluted proteins were loaded on an SDS-PAGE followed by in gel digestion and analysis by MALDI-ToF MS/MS [21], [22]. 4) The method proposed worked well for bacterial cells and the DNA-protein complex is formed *in vivo* unlike the method described by Wu et al, 2011 where a PCR amplified DNA is used for DNA protein complex formation. 5) Microarray is not required and therefore the proposed method is cost effective and does not require the knowledge of whole genome sequence. Also, sufficient amount of protein can be obtained by packing an avidin agarose column and binding the DNA protein complex and further elution of the bound proteins. The method is schematically described in Fig 1.

### Isolation of DNA binding proteins

In the present study, the method is used to isolate the proteins that bind to the *dsz* promoter in a biodesulfurizing bacterium, *Gordonia* sp. IITR100. In desulfurizing bacteria, the desulfurizing genes (in the order *dszA*, *-B* and–*C*) are present as an operon under the control of the *dsz* promoter. The proteins which bind to the *dsz* promoter have not been reported to date [23]. Here, we have employed *in vivo* cross-linking of proteins with DNA followed by affinity capture method to isolate the proteins that specifically bind to the DNA of our interest.

### *In vivo* formaldehyde cross-linking and its reversal

Formaldehyde was used for crosslinking of DNA protein complex [24, 25]. An exponentially growing culture of *Gordonia* sp. IITR100 with an OD_600_~1 was selected as the cells are transcriptionally most active and majority of the cells would have been in actively transcribing state [26].After crosslinking, the cells were harvested, sonicated and the supernatant was collected. An aliquot of this supernatant was digested with T5 exonuclease and was passed through the streptavidin column containing biotinylated primer (PS19) specific to the *dsz* promoter. The proteins bound to the DNA were collected as mentioned above. The formaldehyde crosslinked proteins were eluted because of low pH as it is known that formaldehyde crosslinking is highly dependent on pH [27].

**Fig 2:**
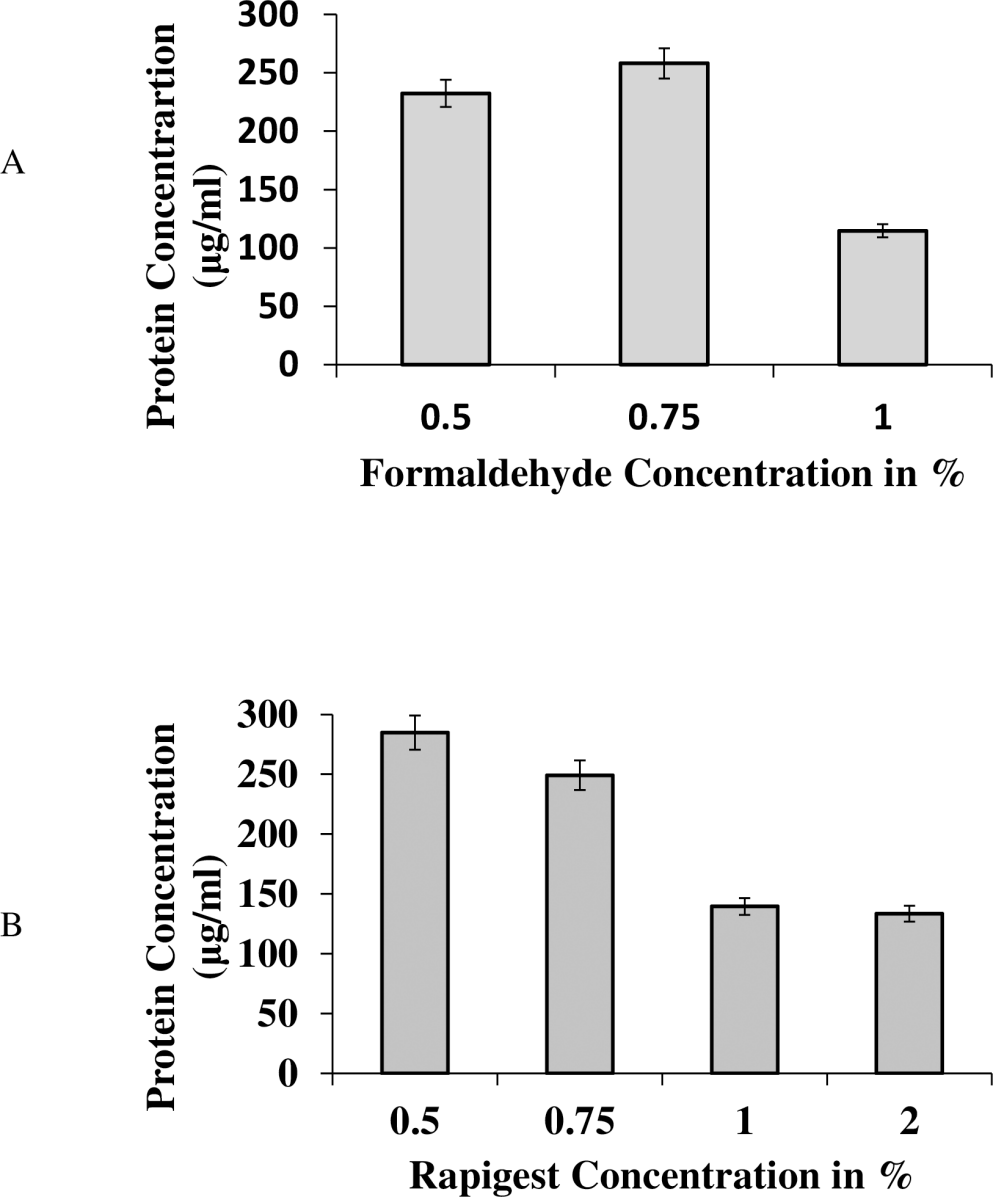
Graph depicting the concentration of protein in eluates when different amount of formaldehyde is used for cross linking (A) and Rapigest for reversal of crosslinking (B).

To get optimal yields various parameters such as concentration of formaldehyde and Rapigest were optimized. Out of the three different concentrations of formaldehyde used (0.5%, 0.75% and 1%), the highest band intensity of eluted proteins was observed at a concentration of 0.75%, therefore this concentration of formaldehyde was used further. The concentration of eluted proteins as determined by Bradford assay is shown in the Fig 2A.

As a control experiment, the cross linking was reversed by adding Rapigest before harvesting the cells. SDS could not be used because it can interfere with MALDI [19]. Four different concentrations of Rapigest 0.5, 0.75, 1 and 2% were used (Fig 2B). After reversal of cross linking, similar protocol as mentioned above was followed to isolate the proteins bound to the DNA. Low Rapigest gave more protein because reversal is not complete and all the proteins remain bound to the DNA that is eluted based on pH. On the other hand, high concentration of Rapigest results in low concentration of proteins because the reversal is complete and protein-DNA complex is not available, instead free DNA is available for binding. Thus, only few proteins were eluted. For maximum reversal 1% rapigest was used in the experiment.

### Characterization of DNA-binding Proteins with *in vivo* cross-linking

**Fig 3:**
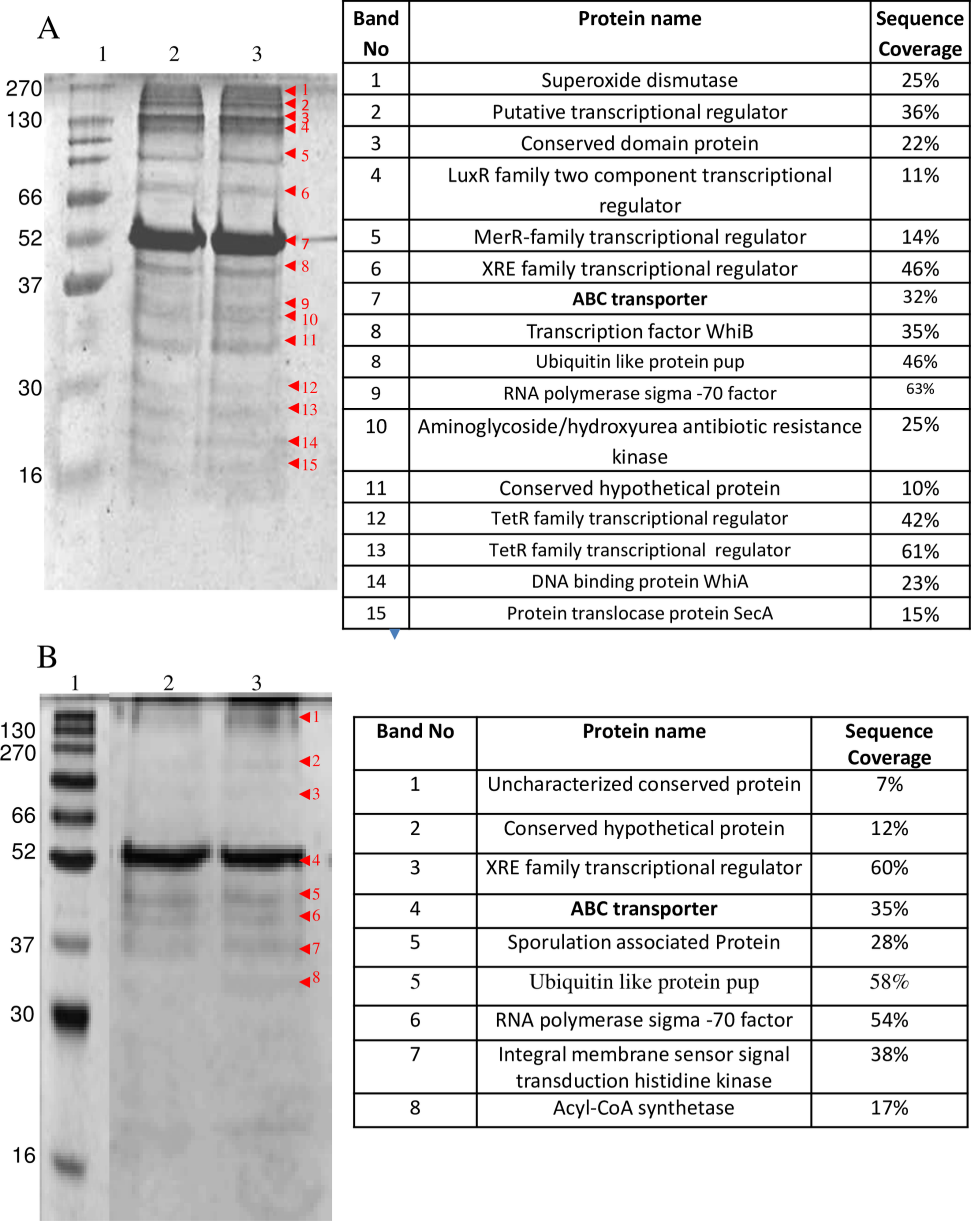
A 12% SDS-PAGE gel showing different elution fractions after crosslinking by formaldehyde and affinity assay (A) and affinity assay after reversal of crosslinking by Rapigest (B). Lane1: broad range protein marker, lane 2–3: elution fractions. The proteins identified and sequence coverage corresponding to each band is shown.

**Fig 4:**
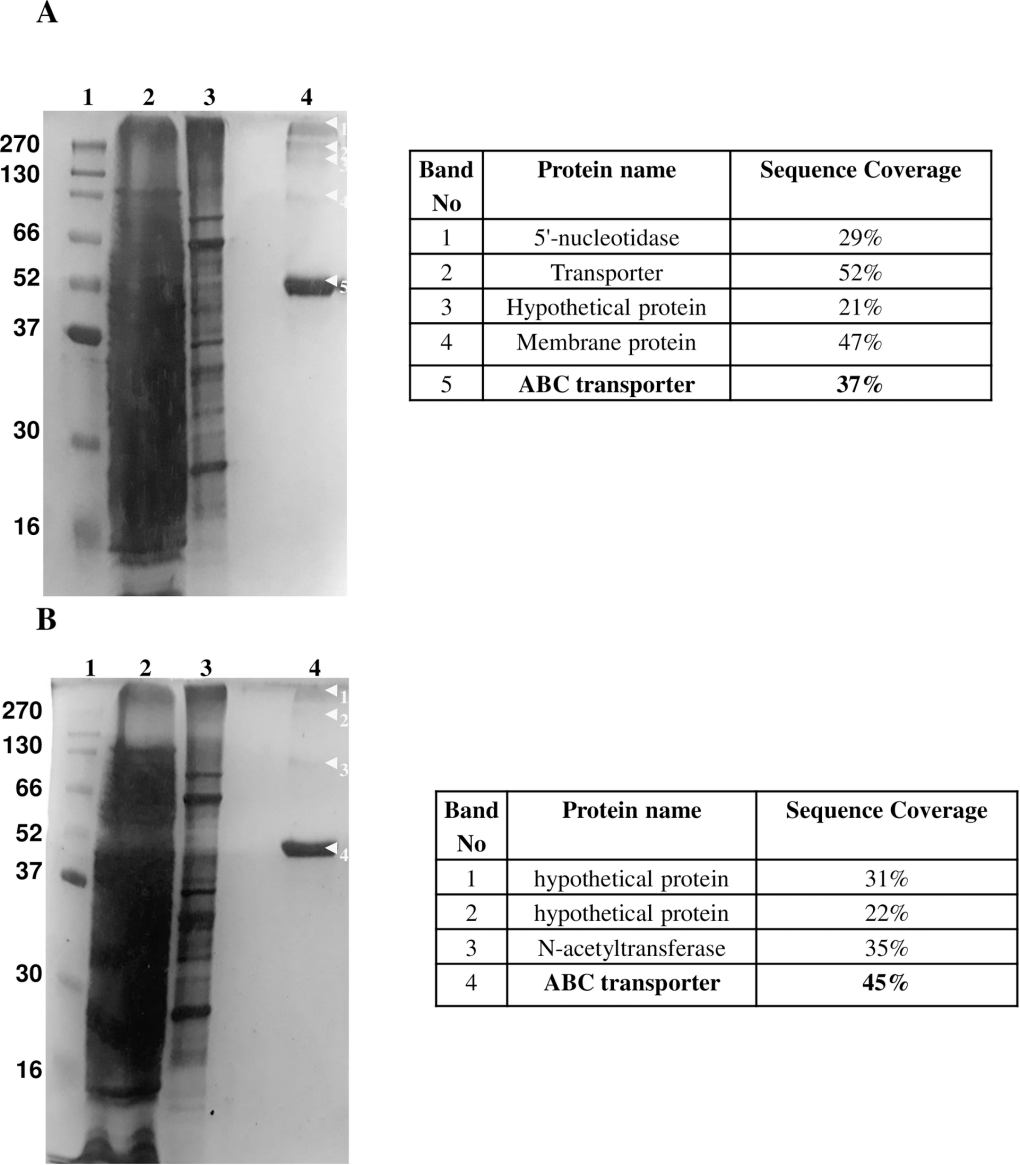
A 12% SDS-PAGE gel showing different elution fractions after crosslinking by formaldehyde and affinity assay. (A) The affinity assay was performed using biotinylated primer specific to the *dsz* promoter attached to streptavidin coated dynabeads. The crosslinked crude extract was passed through the above column without digestion with T5 exonuclease. (B) The affinity assay was performed without attaching any oligonucleotide to streptavidin coated dynabeads. The crosslinked crude extract was digested with T5 exonuclease, then passed through the above column and the different fractions were collected. Lane1: broad range protein marker, lane 2: crude protein extract, lane 3: flow through, lane 4: elution fraction. The proteins identified and sequence coverage corresponding to each band is shown.

**Fig 5:**
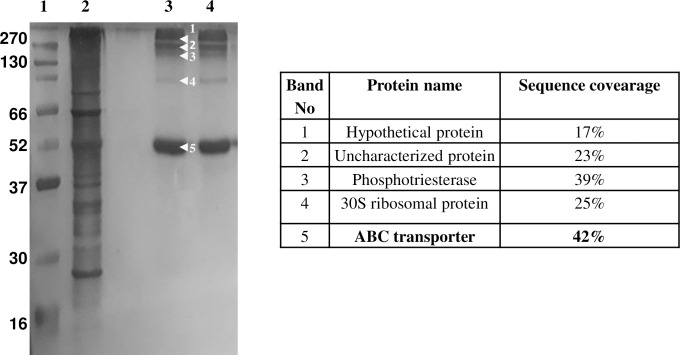
A 12% SDS-PAGE gel showing different elution fractions after crosslinking by formaldehyde and affinity assay. The affinity assay was performed using biotinylated primer of kanamycin promoter attached to streptavidin coated dynabeads. The crosslinked crude extract was digested with T5 exonuclease, then passed through the above column and the different fractions were collected. Lane1: broad range protein marker, lane 2: flow through, lane 3–4: elution fractions. The proteins identified and sequence coverage corresponding to each band is shown.

The eluates from both the experiments (cross linked and reversal of cross linking) were run on 12% SDS PAGE. The number of bands observed by silver staining was 15 in case of eluates from crosslinked samples whereas after reversal of crosslinking the number of bands reduced to 8. The bands were in the size range of 10 to 270 kDa (Fig 3A & 3B). In control experiments where supernatant without digestion with T5 exonuclease was used, about 5 bands were observed (Fig 4A). In another control experiment where beads without bound oligonucleotides were used, 4 bands were observed (Fig 4B). To determine the specificity of the method, a non-specific DNA, biotinylated primer of kanamycin promoter was used. Only few proteins were detected because kanamycin promoter is an *E*. *coli* promoter and is absent in *Gordonia*. It is likely that the absence of complementarity to the primer of kanamycin promoter resulted in detection of fewer proteins (Fig 5).

**Fig 6:**
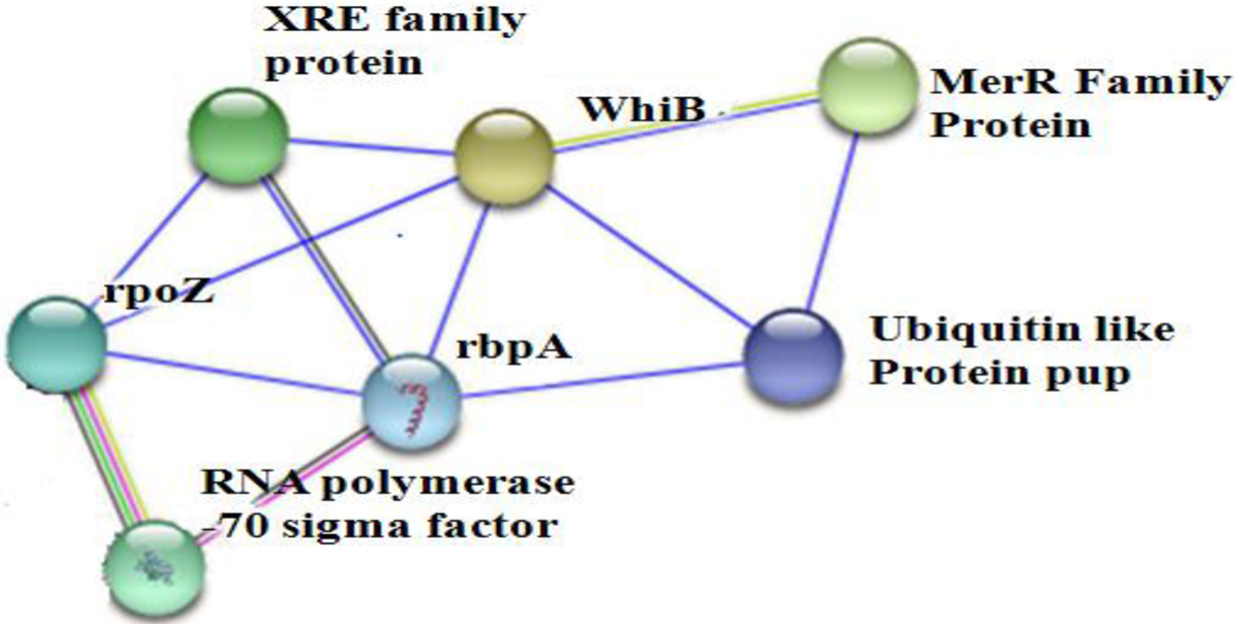
Interactome of proteins obtained after affinity chromatography. XRE family protein, WhiB, MerR family protein, Ubiquitin like protein pup, RNA polymerase -70 sigma factor, rpoZ (transcriptase subunit omega) and rbpA (RNA polymerase binding protein).

The bands obtained in the elution lane of each of the experiments were cut, in gel digested and processed for MS/MS analysis. Based on the Mascot database search, we could identify a transcriptosome complex. On string analysis, we found that a number of identified proteins form an interactome, out of which 3 proteins were found to directly interact with RNA polymerase subunit omega (Fig 6).

### XRE family transcription regulator binds to *dsz* promoter

**Fig 7:**
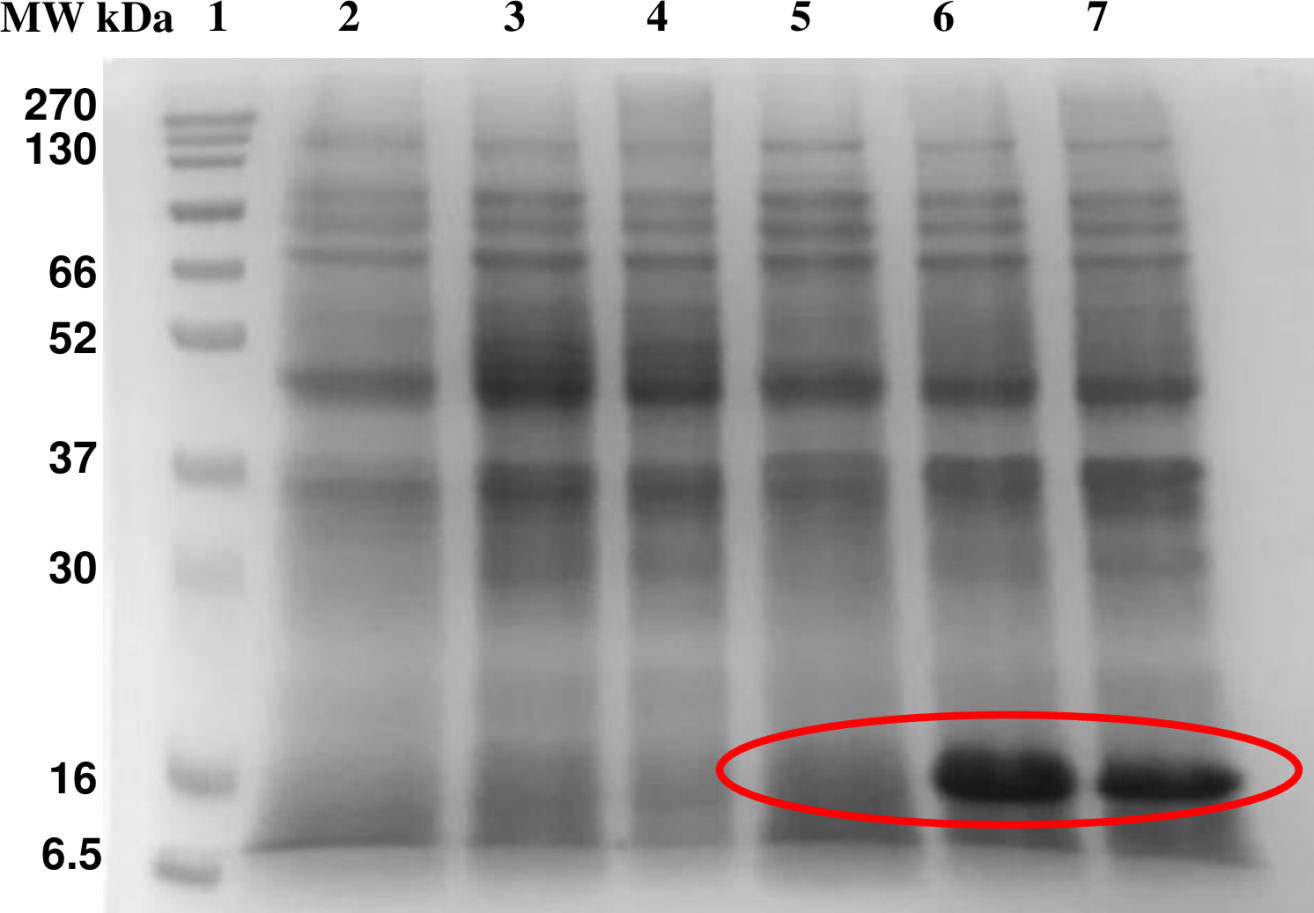
A 12% SDS-PAGE gel showing overexpression of our protein in different expression strains. Lane1: marker, lane2: BL21(DE3) uninduced, lane3: BL21(DE3) 5hr induction, lane4: BL21(DE3) overnight induction, lane5: BL21(DE3) pLysS uninduced, lane6: BL21(DE3) pLysS 5hr induction, lane7: BL21(DE3) pLysS overnight induction.

**Fig 8:**
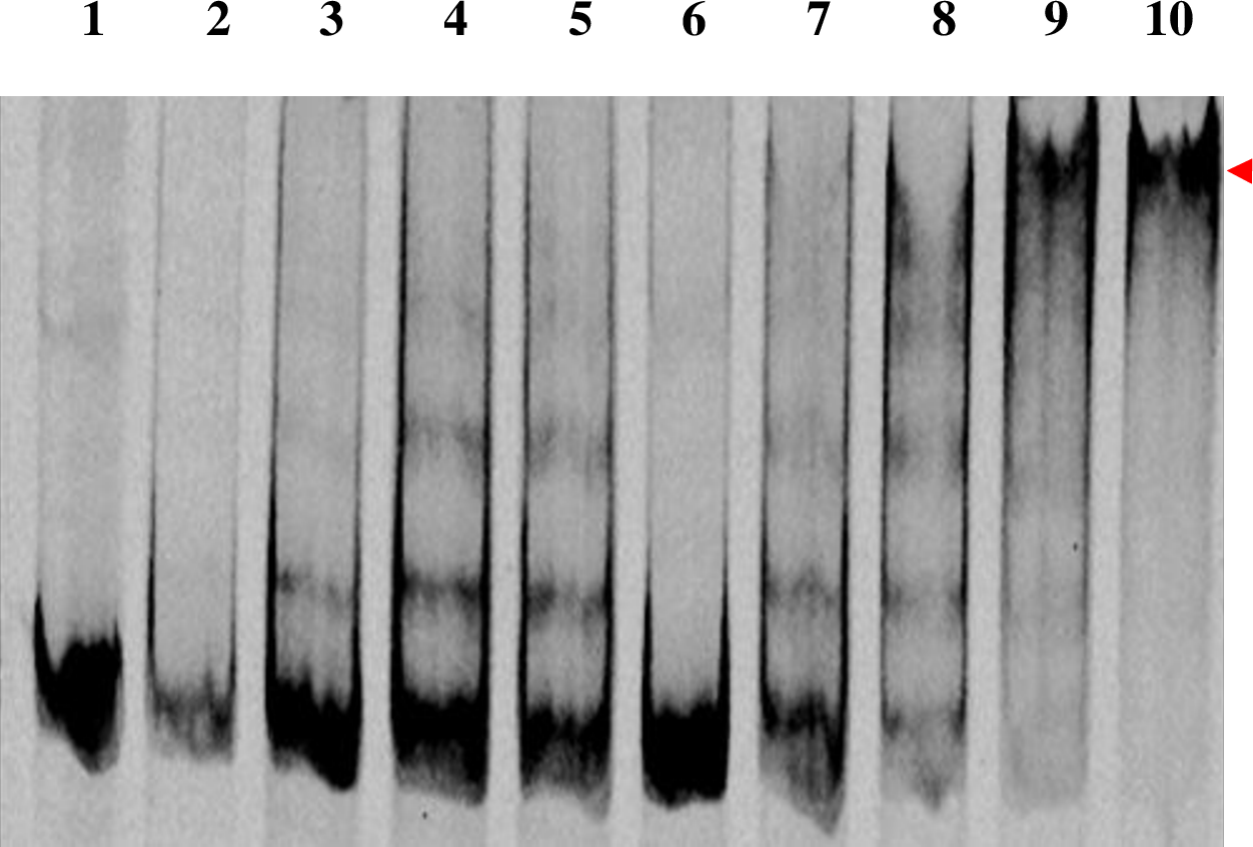
Gel shift assay with partially purified XRE family transcription regulator. Lane 1: free Cy5 labeled *dsz*, lane 2–5: Cy5 labeled *dsz* + partially purified XRE (1, 2, 3,4 μg respectively), lane 6: control lane (it contains other proteins of partial purified lane except XRE family protein), lane 7–10: Cy5 labeled *dsz* + partially purified XRE concentrated (5, 10, 15,20 μg respectively).

To validate the method, one of the genes encoding for XRE like protein obtained in the above assay was selected. The XRE family of transcription regulators is the second most abundant family of transcription regulators in bacteria. It is known for regulating diverse processes in bacteria such as metabolism of nitrogen and carbon containing compounds, lysogeny etc. The XRE family transcription regulator contains a helix turn helix motif which is common in DNA binding proteins [28, 29]. The reason for choosing XRE family transcription regulator is: 1) XRE family transcription regulator was obtained in the elution fractions from both crosslinked and after reversal of crosslinking, although the concentration was reduced after reversal, 2) The gene encoding for XRE family transcription regulator is located at a distance of 8–10 kb from the *dsz* operon in all desulfurizing strains [14]. Thus, to determine whether it binds to the promoter DNA an expression vector pPM4 was used. The expression of XRE family transcription regulator was observed in *E*. *coli* BL21(DE3) pLysS cells (Fig 7). Gel shift assay was performed using Cy5 labeled *dsz* promoter [30] and partially purified XRE (37% pure). Elution fraction which did not contain XRE (or it might be present at a very low concentration which is not visible in the SDS PAGE gel) when used, did not give any shift. At protein concentrations 5 and 10 μg two shifted bands were observed, whereas on increasing the protein concentration further, free DNA disappeared and a major more retarded band was observed (Fig 8).

## Conclusions

The method developed in the present study can be used to isolate unknown proteins that bind to known DNA sequences under *in vivo* conditions from bacterial cells. It confers advantage over other existing methods as it allows the DNA-protein complex formed *in vivo* to be isolated by a simple affinity-based method. Unlike other method as reported by Wu et al [19], microarray is not required thereby making this method cost effective. Moreover, knowledge of whole genome sequence of the bacteria or the protein sequence (as required in ChiP) is not required [8]. Also, sufficient amount of protein can be obtained by packing an avidin agarose column and binding the DNA protein complex and further elution of the bound proteins. Thus, the method is reliable, simple and cost effective for the isolation and identification of DNA-binding proteins. Using the method, we have isolated a transcription regulator XRE, which binds to the *dsz* promoter. This is the first report on the isolation of a transcription regulator which binds the *dsz* operon.

## Supporting information

S1 Fig Purification of XRE. A) Lane 1: marker, lane 2: sonicated supernatant overexpressing XRE, lane 3: flow through, lane 4: washing, lane 5–7: elution fraction with pH 6.5, 5.5 and 4.5 respectively.
(TIF)
