# Supplementary figures and images for: An improved method for the isolation and identification of unknown proteins that bind to known DNA sequences by affinity capture and mass spectrometry
Source: PLoS One. 2018 Aug 23;13(8):e0202602. doi: 10.1371/journal.pone.0202602 (PMC6107227; doi:10.1371/journal.pone.0202602)

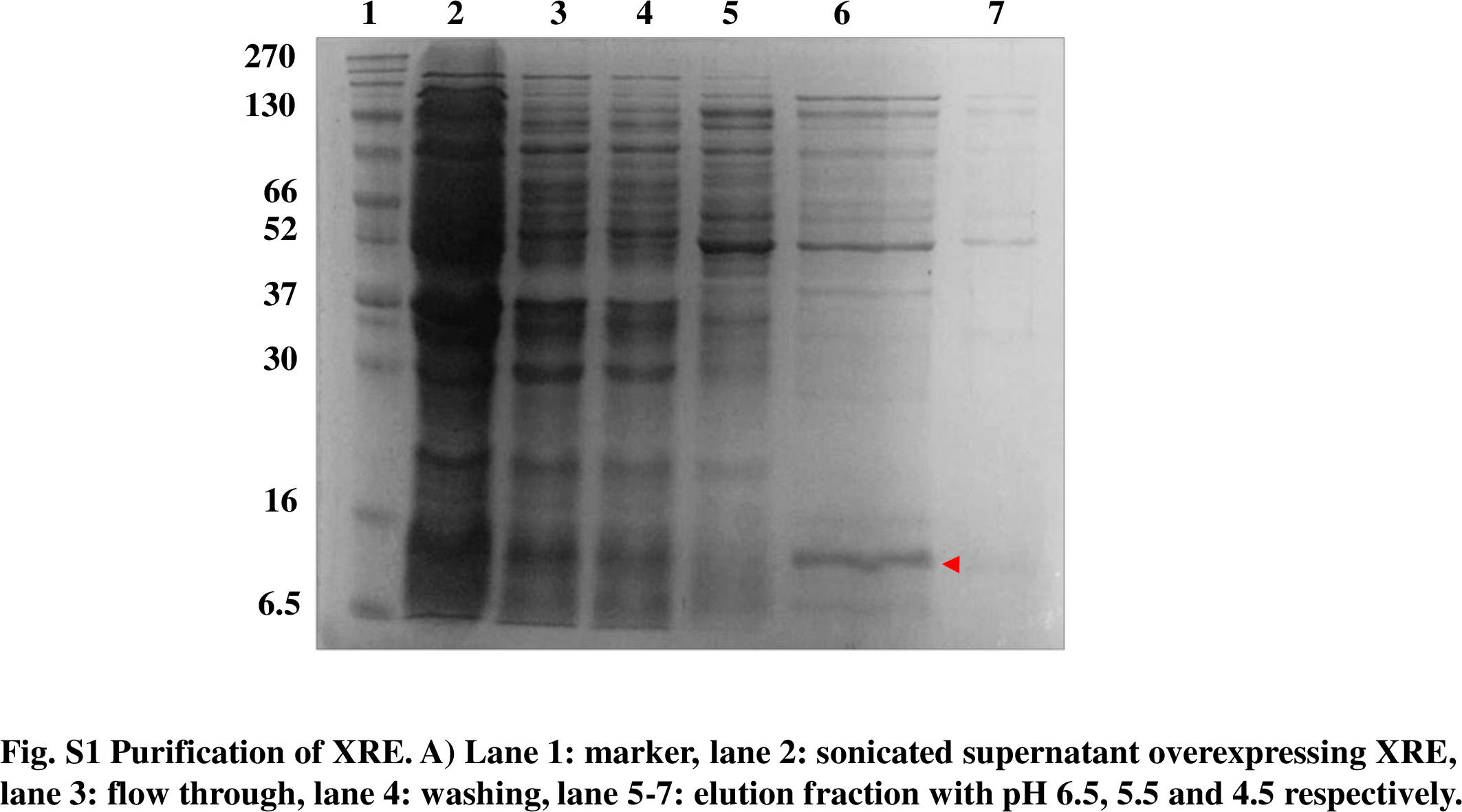

Supplement: S1 Fig — A) Lane 1: marker, lane 2: sonicated supernatant overexpressing XRE, lane 3: flow through, lane 4: washing, lane 5–7: elution fraction with pH 6.5, 5.5 and 4.5 respectively. (TIF) [file pone.0202602.s001.tif]
